# Semiochemical inhibitors for protecting conifers from bark beetles (Coleoptera: Curculionidae) in the Western United States: research, development, and application

**DOI:** 10.3389/finsc.2026.1939049

**Published:** 2026-09-10

**Authors:** Christopher J. Fettig, Jackson P. Audley

**Affiliations:** 1 Pacific Southwest Research Station, USDA Forest Service, Woodland, CA, United States; 2 Department of Plant Sciences, University of California, Davis, Davis, CA, United States

**Keywords:** antiaggregation pheromones, *Dendroctonus*, *Ips*, nonhost volatiles, tree protection

## Abstract

In the western U.S., the research, development and application (RD&A) of semiochemical inhibitors for protecting conifers from bark beetles has focused on antiaggregation pheromones and, to a lesser extent, nonhost volatiles. The sequence begins with identifying potential inhibitors by searching relevant literature; sampling the volatile profiles of bark beetle extracts, frass, infested host materials, hosts, nonhosts, and the forest environment; and/or conducting gas chromatographic-electroantennographic detection analyses of bark beetle antennae. Promising inhibitors are then evaluated for levels of inhibition in trapping and tree protection assays under field conditions. Additional assays are required to refine inhibitors and to inform the spacing and distribution of release devices. Factors affecting levels of tree protection include (a) the population density of the target bark beetle species, (b) forest structure and composition, (c) variability in bark beetle responses to inhibitors, (d) the stereochemistry and chemical stability of inhibitors in the forest environment, (e) release rates of inhibitors, (f) strength of inhibition, (g) zone of inhibition, and (h) the presence of other stressors and disturbances. Once an inhibitor has been successfully developed, there are substantial costs and data requirements associated with registration. The commercial availability of inhibitors for protecting conifers from bark beetles is limited but evidence suggests there are favorable benefit-to-cost returns to be realized with additional investments in RD&A. Opportunities include (a) developing new (or better) inhibitors, inhibitory blends, and formulations, (b) automating (e.g., via unmanned aerial vehicles) and optimizing applications of inhibitors in the field, (c) developing cheaper and more efficient means of synthesizing inhibitors, (d) streamlining regulatory processes and incentivizing RD&A to expedite the commercial availability of products, and (e) integrating inhibitors with other management tactics. We review recent advances in the use of inhibitors for western pine beetle, *Dendroctonus brevicomis* LeConte, southwestern pine beetle, *D*. *barberi* Hopkins, mountain pine beetle, *D*. *ponderosae* Hopkins, Douglas-fir beetle, *D*. *pseudotsugae* Hopkins, spruce beetle, *D*. *rufipennis* (Kirby), northern spruce engraver, *Ips perturbatus* (Eichhoff), and pine engraver, *I*. *pini* (Say). Research on these and other bark beetles has informed the RD&A of inhibitors for other forest and agricultural pests.

## 1 Introduction

Bark beetles (Coleoptera: Curculionidae) are important disturbance agents in conifer forests in the western U.S. Many species prefer colonizing larger-diameter hosts in dense stands with a high proportion of hosts (1). However, there are exceptions. For example, infestations of pine engraver, *Ips pini* (Say), in ponderosa pine, *Pinus ponderosa* Dougl. ex Laws., are negatively correlated with the diameter of *P*. *ponderosa* (2). Tree mortality from bark beetles influences the function, structure, and composition of forests. At low population levels, small gaps in the forest canopy are created by bark beetles colonizing and killing individual trees or small groups of trees stressed by age, drought, defoliation, or other factors. During outbreaks, large amounts of tree mortality may occur over extensive areas.

Several notable bark beetle outbreaks occurred during the early 21^st^ century in the western U.S. Since 2000, mountain pine beetle, *Dendroctonus ponderosae* Hopkins, has impacted >10 million ha (3). Populations peaked in 2007 when ~3.7 million ha were impacted across several states. Based on a network of 125 monitoring plots in five states (Colorado, Idaho, Montana, Utah, and Wyoming), significant reductions in mean tree diameter (by 5.3%), mean tree height (by 15.9%), mean number of trees (by 40.8%), mean basal area of trees (by 52.9%), and mean stand density index (by 51.8%) occurred in the more heavily impacted areas (4). In California, U.S., record levels of tree mortality in the central and southern Sierra Nevada during 2012–2015 prompted the governor to declare a state of emergency (5). Most of the mortality was attributed to western pine beetle, *D*. *brevicomis* LeConte, colonizing *P*. *ponderosa* (6). In Alaska, U.S., an outbreak of spruce beetle, *D*. *rufipennis* (Kirby), has impacted >650,000 ha since 2016 and is the largest in Alaska since the 1990s when ~1.2 million ha were impacted (7). In Colorado, U.S., an outbreak of *D*. *ponderosae* in *P*. *ponderosa*, and to a lesser extent lodgepole pine, *P*. *contorta* Dougl. ex Loud., has impacted tens of thousands of ha, prompting the governor to create a task force to address the outbreak (8).

Host searching, host selection, and host colonization are complex processes in bark beetles. In general, bark beetles must detect and locate the correct habitat, correct host tree species, and the most susceptible hosts (9). If the tree is accepted by the beetle, the beetle bores through the bark and initiates gallery construction in the phloem upon which some species release aggregation pheromones. Successful colonization of living hosts requires overcoming formidable tree defenses (10), which in the case of vigorous hosts is accomplished by recruiting large numbers (hundreds to thousands) of conspecifics to mass attack the tree and overwhelm its defenses. Some bark beetle species release antiaggregation pheromones during the later stages of host colonization to reduce competition within the host (9, 11).

Several tools and tactics are available for managing bark beetles. Suppression addresses current infestations and includes the use of sanitation harvests, insecticides, semiochemicals (attractants and inhibitors) or other treatments (12). Suppression is most effective and cost efficient when applied during the early stages of an infestation. Prevention reduces the susceptibility and vulnerability of forests to bark beetles by manipulating stand, forest, and landscape conditions by thinning, prescribed burning, or other means of altering tree age classes, structures, and compositions (12). The use and timing of suppression and prevention are based on a variety of factors including resource availability, logistical constraints, environmental concerns, the social acceptability of proposed treatments, and the projected impacts of bark beetle infestations on ecosystem goods and services (13).

We review the RD&A of semiochemical inhibitors (hereafter “inhibitors”) for protecting conifers from bark beetles in the western U.S. The genesis of this review was an invitation from the Guest Editors to speak in a symposium “Innovative Research and Development of Insect Repellents” at the 2025 Entomological Society of America Meeting in Portland, Oregon, U.S.

## 2 Inhibitors

### 2.1 Relevant definitions

An *attractant* is a chemical that causes movement towards the chemical. A *repellent* is a chemical that causes movement away from the chemical in the absence of an attractant (14). An *inhibitor* is a chemical that reduces responses to an attractant. In many bark beetle studies, repellency is not demonstrated due to the presence of baits. We agree with Frühbrodt et al. (15) on the importance of the distinction in terminology between repellents and inhibitors, which we have violated in our work (e.g., 16). The literature is replete with such examples.

Throughout this review we use the terms *efficacy* and *effective* to describe inhibitors that cause a statistically significant reduction in attraction to baited traps and/or levels of tree mortality compared to a control. One inhibitor is considered more efficacious (or effective) than another inhibitor if there is a statistically significant difference in attraction or levels of tree mortality between them, or between one inhibitor and the control but not the other inhibitor and the control.

### 2.2 Active ingredients

RD&A has focused on antiaggregation pheromones and, to a lesser extent, nonhost volatiles (NHVs). The most commonly studied antiaggregation pheromones are verbenone for pine bark beetles, which is considered a universal inhibitor by some authors (15), and 3-methylcyclohex-2-en-1-one (methylcyclohexanone, MCH) for Douglas-fir beetle, *D*. *pseudotsugae* Hopkins, and *D*. *rufipennis* (17). It is assumed that both verbenone and MCH reduce intraspecific competition by minimizing overcrowding of developing brood within the host tree. Verbenone is produced by several pathways including autoxidation of the monoterpene α-pinene in the absence of bark beetles and their associates (yeasts and microbes; 18). As such, Lindgren (19) and Lindgren et al. (20) proposed that verbenone is also an indicator of declining host tissue quality, which agrees with its function as an inhibitor or antifeedant for species that do not produce verbenone (e.g., 21).

Bark beetles encounter host trees, nonhost trees, and other nonhost plants when flying. Both host volatiles and NHVs play important roles in host searching and host selection for bark beetles (22, 23). A recent study in *P*. *contorta* forests in the Intermountain West, U.S. found the abundance of nonhost trees had a negative effect on levels of *P*. *contorta* mortality attributed to *D*. *ponderosae* (24), a relationship that likely extends to other bark beetles and forest types. The most commonly studied NHVs are green leaf volatiles (i.e., six-carbon alcohols, aldehydes and related esters; 23) that are used in defense and phytohormone responses (25). Larger groupings of NHVs often result in higher levels of inhibition (23).

Allomonal inhibition, whereby the presence of an aggregation pheromone of one species inhibits another species, has been demonstrated in some bark beetles. For example, components of the aggregation pheromone of California fivespined ips, *I*. *paraconfusus* Lanier, inhibit attraction of *I*. *pini* (26) and red turpentine beetle, *D*. *valens* LeConte (27). There is a risk of trading one source of tree mortality with another source of tree mortality with allomonal inhibition. As such, only limited research has occurred on allomonal inhibitors for mitigating tree mortality attributed to bark beetles.

### 2.3 Products

Information on products registered in each state is available online. For example, the California Department of Pesticide Regulation’s Product/Label database (https://apps.cdpr.ca.gov/docs/label/labelque.cfm) contains information on all pesticide products registered in California including the registrant’s name and address, active ingredients, site/pest category uses, pesticidal type, formulation code, and registration status. A search for “bark beetle” (20 March 2026) yielded two products containing verbenone, SPLAT Verb (EPA Reg. No. 80286-20, ISCA Inc., Riverside, CA, U.S.) and Synergy Shield Verbenone (EPA Reg. No. 90515-2, Synergy Semiochemical Corp., Delta, British Columbia, Canada). No products containing MCH are registered in California, which is not surprising given the limited impacts of *D*. *pseudotsugae* and *D*. *rufipennis* in California. However, 11 states in the western U.S. have at least one MCH product registered. To our knowledge, no products containing NHVs are registered for bark beetles in any state.

Different devices are used to release inhibitors into the active airspace of forests. The most common are bubblecaps, beads, flakes, pouches, and SPLAT (Specialized Pheromone and Lure Application Technology) (28). Efficacy can vary among products with the same active ingredient(s) due to differences in doses, release rates, semiochemistry, and the distribution of release devices, among other factors (Section 5).

### 2.4 Regulatory processes

The U.S. Environmental Protection Agency (EPA) is charged with the regulation and registration of pesticides sold and distributed in the U.S. in accordance with the Federal Insecticide, Fungicide, and Rodenticide Act (FIFRA) of 1947 (29, 30). FIFRA permits states to regulate pesticides provided that states do not allow for the sale or distribution of pesticides prohibited by EPA. Furthermore, state regulations may only differ from EPA regulations by adding more stringent restrictions to use patterns (31). In the U.S., pesticides are defined as any substance intended to prevent, destroy, repel, or mitigate a pest organism (32). Biopesticides fall into three major classes: biochemical pesticides, microbial pesticides, and plant-incorporated protectants. Biochemical pesticides are defined as naturally occurring substances, or derivatives thereof, that are generally less harmful than conventional pesticides to environmental and human health (29, 30). A key component to biopesticides, and one of the major distinctions from conventional pesticides, is a nontoxic mode of action. Bark beetle attractants (e.g., aggregation pheromones) are exempt from EPA registration while bark beetle inhibitors are regulated as biochemical pesticides (33).

In general, registration of biopesticides is cheaper, faster and requires less data than conventional pesticides. The average registration time is ~18 months (30), however, substantial delays can occur (34). Costs range from ~$2,000–5,000 USD (30). The type of biopesticide, whether the biopesticide represents a new compound or product versus an expanded use of a compound or product registered by EPA, data requirements, completeness of the application, and agency workload affect the registration process. A pre-submission meeting (scheduled with EPA) is recommended to clarify data and labeling requirements (30). Typical data requirements include efficacy trials on target pest(s), toxicology data pertaining to human health impacts, and environmental impact assessments evaluating effects on non-target organisms. Once all data are collected, a complete application and associated fees are submitted to EPA and the review process begins. If the biopesticide is approved, additional monitoring may be required during when the biopesticide can be used in accordance with the label (30).

Costs and efforts associated with data collection and registration at EPA are barriers to bringing new bark beetle inhibitors to market. In addition, some states impose additional costs and restrictions (31, 35). Despite efforts to improve, streamline, and clarify regulatory processes (e.g., the 5^th^ Pesticide Registration Improvement Renewal Act, enacted in 2022) a recent report revealed issues stemming from inefficient systems for data exchange, inconsistencies among reviewers and reviewing teams, a lack of transparency for registrants throughout the process, and inefficient communication with outside agencies who’s consultation is often required to complete reviews (34). The IR-4 Biopesticide and Organic Support Program, a U.S. Department of Agriculture (USDA) initiative, exists to assist with the development and registration of biopesticides for specialty and minor crops in the U.S. (36).

## 3 Developmental sequence and study designs

The first step in the RD&A of inhibitors requires identifying potential inhibitors (**Table 1**). This is achieved by searching literature; sampling the volatile profiles of bark beetle extracts, frass, infested host materials, hosts, nonhosts, and the forest environment; and/or conducting gas chromatographic-electroantennographic detection analyses (GC-EAD) of bark beetle antennae. Searching literature is most efficient and cost effective. Some early research on NHVs was performed without prior knowledge of the sensory perception of NHVs in the target bark beetle species (e.g., via data from GC-EAD studies) based on the assumption that bark beetles should be able to detect nonhost cues. This turned out to be a valid assumption. Some researchers have been critical of this approach, as well as with evaluating potential inhibitors that do not have a strong coevolutionary basis for selection (e.g., NHVs from plants not sympatric with the target bark beetle species). We disagree with these ascertains given the overall goal is to develop novel inhibitors with efficiency.

**Table 1:** Steps in the RD&A of semiochemical inhibitors for protecting conifers from bark beetles in the western United States.

Steps	Methods	Commitments
1. Identify potential inhibitor(s)	Search relevant literature. Sample the volatile profiles of bark beetle extracts, frass, infested host materials, hosts, nonhosts, and the forest environment. Conduct gas chromatographic-electroantennographic detection analyses (GC-EAD) to determine if the target bark beetle species can detect the semiochemical.	Days to weeks Months Months
2. Evaluate potential inhibitors in baited trapping assays	Conduct baited trapping assays to determine levels of inhibition in the target bark beetle species. Assays should be repeated in different locations, bark beetle populations, and times of year. Inhibitor(s) that cause >90% reductions in trap catches have promise for Step 4.	Months to years
3. Optimize levels of inhibition in baited trapping assays	Conduct baited trapping assays to optimize doses, release rates, and combinations of inhibitors. Assays should be repeated in different locations, bark beetle populations, and times of year. Inhibitor(s) that cause >90% reductions in trap catches have promise for Step 4.	Months to years
4. Evaluate potential inhibitors for protecting individual host trees	Conduct individual tree protection studies to determine efficacy. Studies should be repeated in different locations, bark beetle populations, and hosts (if applicable). In most studies, host trees should be baited to help ensure experimental trees are challenged by the target bark beetle species. Inhibitor(s) that significantly reduce levels of tree mortality compared to the baited control have promise for Step 6.	Years
5. Determine the zone of inhibition provided by release of the most promising inhibitor(s) identified in Step 4	Conduct zone of inhibition trapping assays (see 49 for common design) and/or examine effects of inhibitors on neighboring trees.	Weeks to years
6. Evaluate potential inhibitors for protecting stands	Conduct small-scale stand studies to determine levels of tree protection. Results from Step 5 can be used to determine spacing of inhibitor(s). In most studies, the center of each experimental plot should be baited to help ensure inhibitors are challenged by the target bark beetle species.	Years
7. Registration and commercialization	Commercialization of inhibitors involves substantial risks and investments. There are fees for registration and other costs associated with environmental and human health testing requirements.	Years

**Figure 1:**
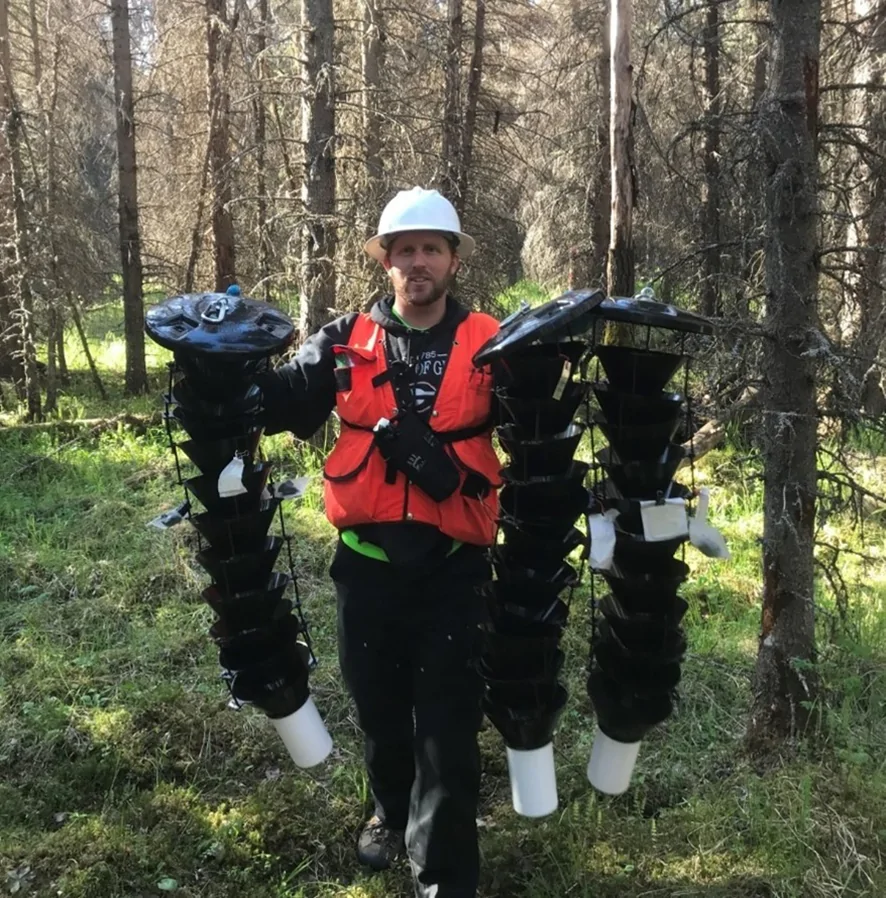
Once potential inhibitors have been identified, levels of inhibition are evaluated in baited trapping assays. Common experimental designs include completely randomized (CRD) or randomized complete block (RCBD) designs. Sufficient sample sizes are obtained by using large numbers of traps or by frequent collection of trap catches and subsequent re-randomization of treatments as depicted for *Dendroctonus rufipennis* in Alaska, U.S. Photo by C.J. Fettig, USDA Forest Service.

Once potential inhibitors have been identified, the next steps involve evaluating levels of inhibition in trapping and tree protection assays (**Table 1**). Common experimental designs for trapping assays include completely randomized (CRD) or randomized complete block (RCBD) designs (37). In a CRD, data are viewed as if they were random samples from a normal distribution. Each treatment is randomly assigned among all sample units (traps) (38). The RCBD represents a level of restriction on randomization but allows investigators to reduce residual error by removing variability due to nuisance variables. For bark beetles, these may include site (location), temporal or other factors that can influence bark beetle flight, population densities, or responses to traps. However, we have observed that the RCBD is often used without knowledge of the effects of nuisance variables on trap catches and therefore may unnecessarily sacrifice degrees of freedom error in the process. In a RCBD, each treatment is randomly assigned among the number of sample units within each block, and not the entire sample population as in the CRD (38). Latin square designs have received less attention than the CRD or RCBD in trapping assays but allow for control of two nuisance variables. The row and column of each square represent the two levels of restriction on randomization. Sufficient sample sizes in trapping assays are obtained by using large numbers of traps or by frequent (e.g., daily) collection of trap catches and subsequent re-randomization of treatments for a period of time (**Figure 1**). Inhibitors that cause >90% reduction in trap catches generally hold promise for tree protection (39).

The most common experimental design for tree protection assays is the CRD, although other designs (e.g., paired T-tests and RCBD) are used. Assays are conducted at the individual tree and larger spatial scales (**Table 1**). A challenge with conducting tree protection assays is the costs and efforts associated with establishing, treating, and evaluating treatment effects on large experimental plots, which limits the number of replicates (often *n* < 6) and statistical power. Once an effective inhibitor has been identified, subsequent steps involve additional trapping and tree protection assays to refine the spacing and distribution of release devices, doses and release rates, among other factors (**Table 1**).

## 4 Recent advances in the RD&A of inhibitors

### 4.1 Dendroctonus brevicomis

*Dendroctonus brevicomis* causes substantial mortality of *P*. *ponderosa* in the western U.S., particularly in California and Oregon (40). The only other host in the western U.S. is Coulter pine, *P*. *coulteri* D. Don, a species indigenous to the Transverse and Peninsular Ranges of California. Female *D*. *brevicomis* release an aggregation pheromone, *exo*-brevicomin, during the early stages of host colonization. *exo*-Brevicomin is attractive to males and females, especially in the presence of host terpenes (41). Frontalin, an aggregation pheromone produced by male *D*. *brevicomis* (42), further increases attraction to the host tree (43, 44). During the later stages of host colonization, the antiaggregation pheromones verbenone, *trans*-verbenol, and ipsdienol are produced and inhibit attraction to the host tree (45, 46). A recent review is available on the chemical ecology of *D*. *brevicomis* (47).

A flake formulation of verbenone applied to individual *P*. *ponderosa* was ineffective for protecting *P*. *ponderosa* from *D*. *brevicomis* in California (48). Similarly, verbenone pouches were ineffective for protecting 2-ha experimental plots of *P*. *ponderosa* (49). Fettig et al. (50) reported verbenone inhibited attraction of *D*. *brevicomis* to baited traps by only ~35–48%. It is therefore not surprising that verbenone alone is ineffective for protecting *P*. *ponderosa* from *D*. *brevicomis*, despite verbenone being the primary antiaggregation pheromone of *D*. *brevicomis* (46).

Poland et al. (51) examined the effects of NHVs on *D*. *brevicomis* attraction to baited traps in British Columbia and reported that the green leaf aldehyde (*E*)-2-hexenal, and two green leaf alcohols, (*E*)-2-hexen-1-ol and (*Z*)-2-hexen-1-ol, reduced attraction. Fettig et al. (50) reported that combinations of angiosperm bark volatiles (benzaldehyde, benzyl alcohol, (*E*)-conophthorin, guaiacol, nonanal, and salicylaldehyde), green leaf volatiles ((*E*)-2-hexenal, (*E*)-2-hexen-1-ol, and (*Z*)-2-hexen-1-ol), and the nine compounds combined had no effect on the response of *D*. *brevicomis* to baited traps in California. However, trap catches were reduced to levels significantly below that of verbenone alone when verbenone was combined with the bark and green leaf volatiles. Based on these results, an assay in California was the first to demonstrate the successful application of an inhibitor for protecting *P*. *ponderosa* from *D*. *brevicomis* (52).

Fettig et al. (53) further examined the response of *D*. *brevicomis* to several blends of NHVs and verbenone in baited traps in California with the goal of improving the efficacy of their blend and reducing the number of compounds. They documented the inhibitory effect of a five-compound blend (nonanal, (*E*)-2-hexenal, (*E*)-2-hexen-1-ol, (*Z*)-2-hexen-1-ol, and verbenone). Later, it was found that removal of the aldehydes could be achieved by substituting acetophenone, which was found inhibitory to *D*. *brevicomis* several years earlier (54, 55). Acetophenone is produced by some bark beetles (56) and plants and is approved by the U.S. Food and Drug Administration as a synthetic flavoring in food, beverages, soaps, and lotions. The resulting blend (acetophenone, (*E*)-2-hexen-1-ol + (*Z*)-2-hexen-1-ol, and verbenone, “Verbenone Plus”) has been demonstrated to inhibit the response of *D*. *brevicomis* to baited traps and trees in multiple assays in California and British Columbia (57). Verbenone Plus has not been registered for use in the U.S. (**Table 2**).

**Table 2:** The availability and efficacy of semiochemical inhibitors for protecting conifers from select bark beetle species in the western United States.

Bark beetle species	Host species	Inhibitor^1^	Has efficacy been demonstrated for individual tree protection?	Has efficacy been demonstrated for stand protection?	Is the inhibitor used operationally?^3^	Timing of treatment^4^
*Dendroctonus adjunctus*	*Pinus* spp.	–^2^	–	–	–	–
*Dendroctonus barberi*	*Pinus ponderosa*	Verbenone Plus	No	Yes	No	March and June
*Dendroctonus brevicomis*	*Pinus ponderosa*	Verbenone Plus	Yes	Yes	No	May
*Dendroctonus jeffreyi*	*Pinus jeffreyi*	–	–	–	–	–
*Dendroctonus ponderosae*	*Pinus albicaulis*	verbenone	Yes	Yes	Yes	June
*Dendroctonus ponderosae*	*Pinus contorta*	verbenone	Yes	Yes	Yes	June
*Dendroctonus ponderosae*	*Pinus flexilis*	verbenone	Yes	No	Yes	June
*Dendroctonus ponderosae*	*Pinus lambertiana*	verbenone	Yes	No	Yes	May
*Dendroctonus ponderosae*	*Pinus ponderosa*	–	–	–	–	–
*Dendroctonus pseudotsugae*	*Pseudotsuga menziesii*	MCH	Yes	Yes	Yes	May
*Dendroctonus rufipennis*	*Picea* spp.	MCH	Yes	Yes	Yes	May-June
*Dryocoetes confusus*	*Abies lasiocarpa*	–	–	–	–	–
*Ips confusus*	*Pinus* spp.	–	–	–	–	–
*Ips paraconfusus*	*Pinus* spp.	–	–	–	–	
*Ips perturbatus*	*Picea* spp.	verbenone + conophthorin	Yes	No	No	May
*Ips pini*	*Pinus ponderosa*	–	–	–	–	–
*Scolytus ventralis*	*Abies* spp.	–	–	–	–	–

^1^ Verbenone Plus = acetophenone, (*E*)-2-hexen-1-ol + (*Z*)-2-hexen-1-ol, and verbenone. MCH = 3-methylcyclohex-2-en-1-one.

^2^ = none or not applicable.

^3^ Based on registration in at least one state in the western U.S. on May 5, 2026 and operational use by natural resource managers.

^4^ Approximate timing of treatments based on peer-reviewed literature and experience. For *Dendroctonus barberi*, evidence suggests that three treatments per year may be required during warm years due to the effects of temperature on the release of inhibitors.

Efficacy is based on a statistically significant reduction in levels of mass attacks or tree mortality compared to a control treatment in at least one peer-reviewed study. The bark beetles listed account for most of the tree mortality attributed to bark beetles in the western U. S.

### 4.2 Dendroctonus barberi

Southwestern pine beetle, *Dendroctonus barberi* Hopkins, and *D*. *brevicomis* were synonymous from 1963−2019 but were separated as full species in 2019 (58–60). Before 1963, *D*. *barberi* and *D*. *brevicomis* were full species. Today, *D*. *barberi* is considered geographically isolated from *D*. *brevicomis* by the Great Basin, Mojave, and Sonoran Deserts (59). The primary host is *P*. *ponderosa* and while pinyon pine, *P*. *edulis* Engelm., and Douglas-fir, *Pseudotsuga menziesii* (Mirb.) Franco, have also been recorded as hosts colonization of these species is rare (61). During the early stages of host colonization, female *D*. *barberi* release the aggregation pheromone *endo*-brevicomin (62). Male *D*. *barberi* release the aggregation pheromone frontalin which enhances attraction to the target tree (60, 63). During the later stages of host colonization, the antiaggregation pheromones verbenone and *trans*-verbenol are produced and inhibit attraction to the host tree.

Prior to 2024, RD&A had not occurred on inhibitors for *D*. *barberi*. In response to a large outbreak in the Davis Mountains Preserve in West Texas, U.S., Verbenone Plus was evaluated in trapping and tree protection assays during 2024–2026. Verbenone Plus inhibited the response of *D*. *barberi* to baited traps in all assays, by as much as 99.5%, and was effective for protecting experimental plots of *P*. *ponderosa* from *D*. *barberi* (64). Verbenone Plus was reapplied in 2026 to protect some of the remaining *P*. *ponderosa* at the Davis Mountains Preserve and will provide additional information on the efficacy of Verbenone Plus for *D*. *barberi*. A study led by the Pacific Southwest Research Station is evaluating the efficacy of Verbenone Plus and SPLAT Verb for protecting *P*. *ponderosa* from a complex of bark beetles including *D*. *barberi*, *D*. *ponderosae*, roundheaded pine beetle, *D*. *adjunctus* Blandford, and *Ips* spp. in Colorado. Preliminary results will be available in 2027.

Gomez et al. (64) commented that given the very high levels of inhibition observed for Verbenone Plus in their trapping assays, verbenone alone may be worth evaluating as an inhibitor for *D*. *barberi* in future assays.

### 4.3 Dendroctonus ponderosae

The host range of *D*. *ponderosae* includes at least 15 *Pinus* species. Preferred hosts are *P*. *contorta*, *P*. *ponderosa*, limber pine, *P*. *flexilis* E. James, sugar pine, *P*. *lambertiana* Dougl., and whitebark pine, *P*. *albicaulis* Engelm (65). Occasionally *D*. *ponderosae* colonizes other Pinaceae during outbreaks, including Engelmann spruce, *Picea engelmannii* Parry ex Engelm. Female *D*. *ponderosae* release an aggregation pheromone, *trans*-verbenol, during the early stages of host colonization (66, 67). *exo*-Brevicomin is then released by males and females, and is thought to be attractive at low concentrations (68, 69) and inhibitory at high concentrations (56, 70). *cis*-Verbenol, another aggregation pheromone released by females, increases attraction to the target tree (71), especially in the presence of host terpenes. During the later stages of host colonization, the antiaggregation pheromone verbenone is released by females (72) and inhibits attraction to the host tree. The release of 2-phenylethanol by males (56) and 1-octen-3-ol by females (73) further inhibit attraction.

**Figure 2:**
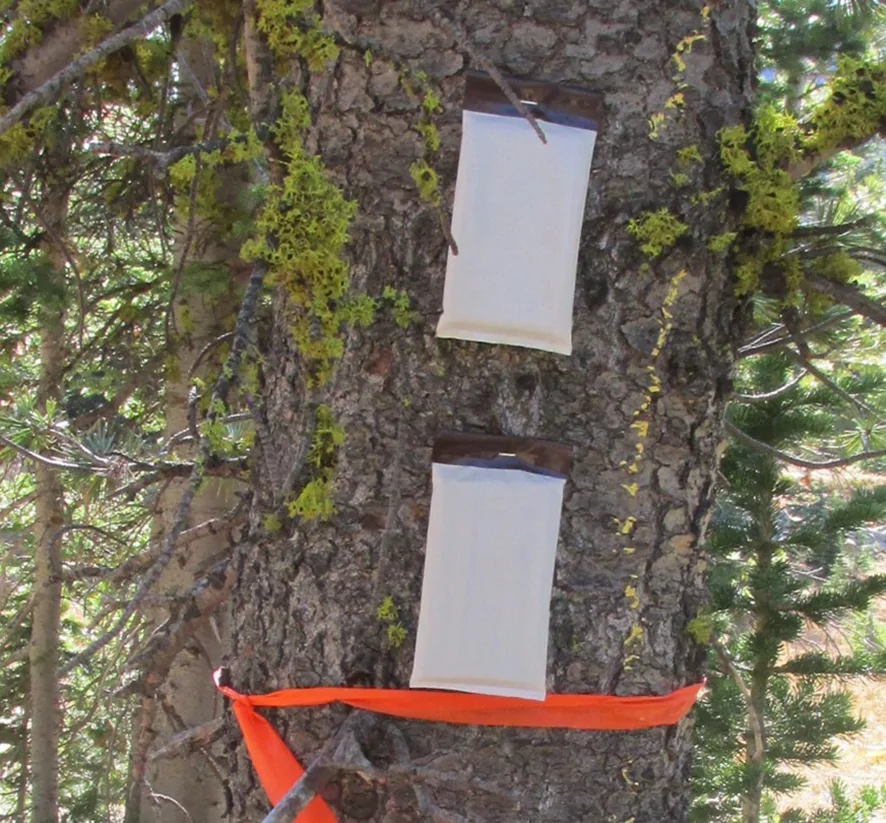
Two verbenone pouches on the bole of *Pinus albicaulis* in Montana, U.S. In 2023, *P*. *albicaulis* was listed as threatened in the U.S. with white pine blister rust, *Dendroctonus ponderosae*, altered wildfire patterns, and climate change identified as major threats to its existence (225). Verbenone pouches, SPLAT Verb, and verbenone flakes have been demonstrated effective for protecting *P*. *albicaulis* from *D*. *ponderosae* in multiple assays. Photo by C.J. Fettig, USDA Forest Service.

More RD&A has focused on inhibitors for *D*. *ponderosae* than any other bark beetle, which is attributed to the large impacts caused by this species (65). The first evidence of inhibition occurred with verbenone in laboratory and baited trapping assays (74). Thereafter, multiple assays demonstrated that verbenone bubblecaps and verbenone pouches (**Figure 2**) were effective for protecting individual *P. contorta* and stands of *P*. *contorta* (e.g., 69, 75–77). Verbenone is also effective for protecting *P*. *flexilis*, *P*. *lambertiana*, and *P*. *albicaulis* from *D*. *ponderosae* (78–82), but is ineffective for protecting *P*. *ponderosa* (**Table 2**), a common host species and the most widely distributed conifer in the U.S. However, data from *P*. *ponderosa* forests are limited, especially for registered formulations of verbenone. Negrón et al. (83) reported that 5-g verbenone pouches applied at 30 and 50 pouches per 0.41-ha had no effect on *D*. *ponderosae* attacks in *P*. *ponderosa* stands in South Dakota, U.S. Fettig et al. (82) found that 70 g SPLAT Verb tree^-1^ was ineffective for protecting individual *P*. *ponderosa* in Montana, U.S., but a confounding effect in their study was that 89% of *P*. *ponderosa* colonized by *D*. *ponderosae* were also colonized by *D*. *brevicomis*, *I*. *pini*, and/or emarginated ips, *I*. *emarginatus* (LeConte). Verbenone is ineffective for protecting *P*. *ponderosa* from *D*. *brevicomis* (Section 4.1), and the effects of verbenone for protecting *P*. *ponderosa* from *I*. *pini* (Section 4.5) and *I*. *emarginatus* are unknown. A study led by Colorado State University is evaluating the efficacy of SPLAT Verb for protecting *P*. *ponderosa* from *D*. *ponderosae* in the Front Range of Colorado. Preliminary results will be available in 2027. The lack of efficacy for verbenone in *P*. *ponderosa* is likely attributed to structural effects (lower tree densities; 79), which influence the concentration and directionality of verbenone plumes in forests (Section 5.2).

Once the inhibitory effect of verbenone was well documented, subsequent research focused on NHVs in hopes of improving levels of tree protection. Wilson et al. (84) found that a combination of (*E*)-2-hexen-1-ol and (*Z*)-3-hexen-1-ol reduced the number of *P*. *contorta* attacked by *D*. *ponderosae* in British Columbia. Huber and Borden (85, 86) conducted assays with different combinations of NHVs and verbenone in British Columbia and reported similar results, but with the most effective treatments combining NHVs with verbenone. Borden et al. (87) reported only 2.1% of *P*. *contorta* were mass attacked on plots treated with NHVs and verbenone compared to 26.6% in the control treatment in British Columbia. Later, Fettig et al. (88) demonstrated that Verbenone Plus was effective for protecting stands of *P*. *albicaulis* in California, where operational treatments of verbenone alone had been unsuccessful. More recently, Fettig and Munson (89) demonstrated that Verbenone Plus and verbenone alone were effective for protecting individual *P*. *contorta* and stands of *P*. *contorta* from *D*. *ponderosae* in Idaho and Utah, U.S. However, verbenone alone was as effective as Verbenone Plus.

In more recent years, RD&A has focused on new formulations of verbenone. Gillette et al. (48, 78, 90–92) demonstrated that verbenone could be deployed in plastic flakes applied dry by aerial application, so that they fall to the forest floor, or with a sticking agent by ground application, so that they adhere to the tree bole. Later research focused on SPLAT Verb, a wax emulsion matrix containing 10.0% verbenone (by weight) applied with caulking guns that usually degrades within a year of application (93, 94). SPLAT Verb was effective for protecting *P*. *contorta*, *P*. *lambertiana*, and *P*. *albicaulis* from *D*. *ponderosae* in assays conducted in California, Idaho, Montana, Oregon, and Wyoming, U.S. (e.g., 80, 82, 89, 94). In some studies, 100% tree protection was reported (94). The high levels of tree protection afforded by SPLAT Verb are attributed to the ability of applying release points (dollops) at high densities, and a larger zone of inhibition than reported for other formulations of verbenone. For example, SPLAT Verb was more effective than the verbenone pouch for protecting *P*. *contorta* stands from *D*. *ponderosae* in Idaho, despite both products being applied at the same dose and similar release rates (94). The higher level of efficacy was attributed, in part, to applying 4X more release points of SPLAT Verb (dollops of SPLAT Verb contained 1.75 g of verbenone) plot^-1^ than pouches (pouches contained 7 g of verbenone).

A verbenone pouch was first registered by EPA in 1999 based on research on southern pine beetle, *D*. *frontalis* Zimmermann (95). Several other formulations and products containing verbenone have been registered since 1999 (28). SPLAT Verb was first registered by EPA in 2013 and is now widely used throughout the western U.S.

### 4.4 Dendroctonus pseudotsugae

*Dendroctonus pseudotsugae* causes substantial mortality of *Ps*. *menziesii* in the western U.S. (40). Occasionally western larch, *Larix occidentalis* Nutt., is colonized during outbreaks (96). Once female *D. pseudotsugae* enter a host tree, they emit frontalin, seudenol and MCOL, the primary aggregation pheromones (97–100). Host monoterpenes (101, 102) and ethanol enhance responses to these aggregation pheromones (103, 104). During the later stages of host colonization, the antiaggregation pheromone MCH is produced by males and inhibits attraction to the host tree (105, 106).

**Figure 3:**
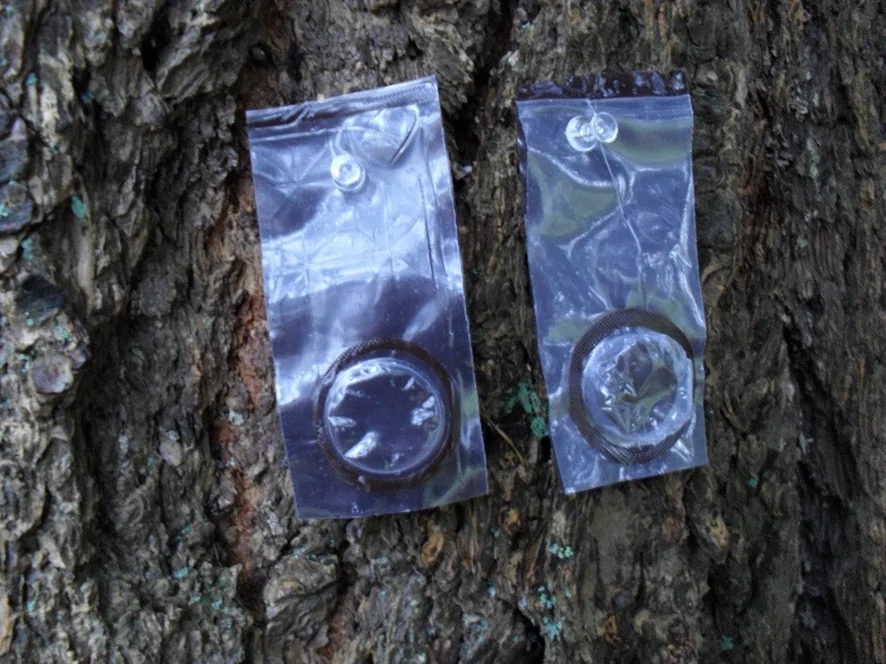
MCH bubblecaps on the bole of *Pseudotsuga menziesii* in Oregon, U.S. The widespread use of MCH bubblecaps to protect *Ps*. *menziesii* from *Dendroctonus pseudotsugae* is an unparalleled success among bark beetle inhibitors. The number of MCH bubblecaps sold annually treats ~8,000 ha (112). Photo by R.A. Progar, USDA Forest Service.

Early RD&A focused on protecting downed *Ps. menziesii* as a means of reducing *D. pseudotsugae* population growth (i.e., populations can rapidly increase in downed *Ps. menziesii* with compromised defenses). A formulation of MCH in dimer acid polyamide beads reduced colonization of downed *Ps. menziesii* in several studies (107–109) but was not registered. Later RD&A focused on protecting live, standing *Ps. menziesii* in Oregon with a bubblecap formulation containing 400 mg MCH released at ~7 mg d^-1^ per bubblecap (104) (**Figure 3**). The percentage of *Ps*. *menziesii* that were mass attacked by *D. pseudotsugae* was significantly lower on MCH-treated plots (0.2%) than control plots (8.5%). Release rates were later optimized to 1.2−1.6 mg d^-1^ and an effective, operational deployment of 75 bubblecaps ha^-1^ was defined (110, 111). Subsequent tree protection assays evaluating higher release rates of MCH and greater spacings among bubblecaps further demonstrated the efficacy of MCH bubblecaps for protecting *Ps. menziesii* from *D. pseudotsugae* (112; **Table 2**). Gillette et al. (113) applied MCH-releasing flakes by helicopter to 4.05-ha plots in Washington, U.S. and reported attack rates in untreated plots were ~10 times greater than in MCH-treated plots.

Bubblecaps are made of two layers of plastic film that degrade very slowly in the environment, requiring removal from the field after use. Applications of MCH often occur in remote, steep terrain, and therefore more degradable formulations of MCH that do not require removal from the field are desirable. Based on the success of SPLAT Verb for *D*. *ponderosae* (Section 4.3), a similar formulation containing 10.0% MCH (by weight) was developed (SPLAT MCH, ISCA Inc., Riverside, CA) for *D. pseudotsugae* (114). In an individual-tree assay in Idaho, MCH bubblecaps and SPLAT MCH significantly reduced the proportion of *Ps*. *menziesii* colonized and killed by *D. pseudotsugae*. In New Mexico, U.S., MCH bubblecaps and SPLAT MCH significantly reduced the proportion of *Ps*. *menziesii* colonized and killed on 0.41-ha experimental plots but in Idaho both MCH treatments had no effect on *D. pseudotsugae* colonization and only MCH bubblecaps reduced levels of *Ps*. *menziesii* mortality (114). A recent study by Ross et al. (115) demonstrated the efficacy of a biodegradable, bead formulation of MCH (Hercon^®^ Bio-Beads™ MCH, Hercon Environmental, Emigsville, PA, U.S.) for protecting 0.4-ha experimental plots in Idaho.

While NHVs have been demonstrated to inhibit the response of *D*. *pseudotsugae* to baited traps (85), research on NHVs for protecting *Ps*. *menziesii* from *D. pseudotsugae* has been limited; presumably due to the high efficacy observed for MCH alone (112). A MCH bubblecap was first registered by EPA in 1999. Several other formulations and products containing MCH have been registered since 1999 (28). SPLAT MCH is currently under review at EPA.

### 4.5 Dendroctonus rufipennis

*Dendroctonus rufipennis* causes significant mortality of *Picea* in the western U.S. Lutz spruce, *Pi*. x *lutzii* Little, a natural hybrid of white spruce, *Pi*. *glauca* (Moench) Voss and Sitka spruce, *Pi*. *sitchensis* (Bong.) Carr., and *Pi*. *glauca* are preferred hosts in Alaska while *Pi*. *engelmannii* is the preferred host in the Rocky Mountains (116). Female *D. rufipennis* initiate host colonization and release frontalin (117, 118), seudenol (119), verbenene (120), and MCOL (121). Male beetles also produce frontalin (118) and to a lesser extent seudenol (122). During the later stages of host colonization, the antiaggregation pheromone MCH is produced by males and females and inhibits attraction to the host tree (108). A recent review is available on the chemical ecology of *D*. *rufipennis* (123).

Shortly following the discovery of MCH, inhibitory effects were demonstrated for *D*. *rufipennis* that included reducing the numbers of *D. rufipennis* caught in baited traps in Idaho (124) and the number of attacks on felled *Pi. engelmannii* in Montana (125). The first evidence of MCH protecting live *Picea* from *D. rufipennis* was not reported until 2003. Holsten et al. (126) observed fewer *Pi.* x *lutzii* were colonized by *D. rufipennis* on plots treated with MCH released from Med-e-Cell devices which are used for chronic drug infusions in humans. Med-e-Cell devices contain a small battery-operated pump and storage reservoir that allowed for timed, measured releases of MCH. Conversely, Ross et al. (127) reported that MCH bubblecaps had no effect on numbers of *Pi. engelmannii* colonized by *D. rufipennis* on 1-ha experimental plots in Utah during a severe outbreak.

**Figure 4:**
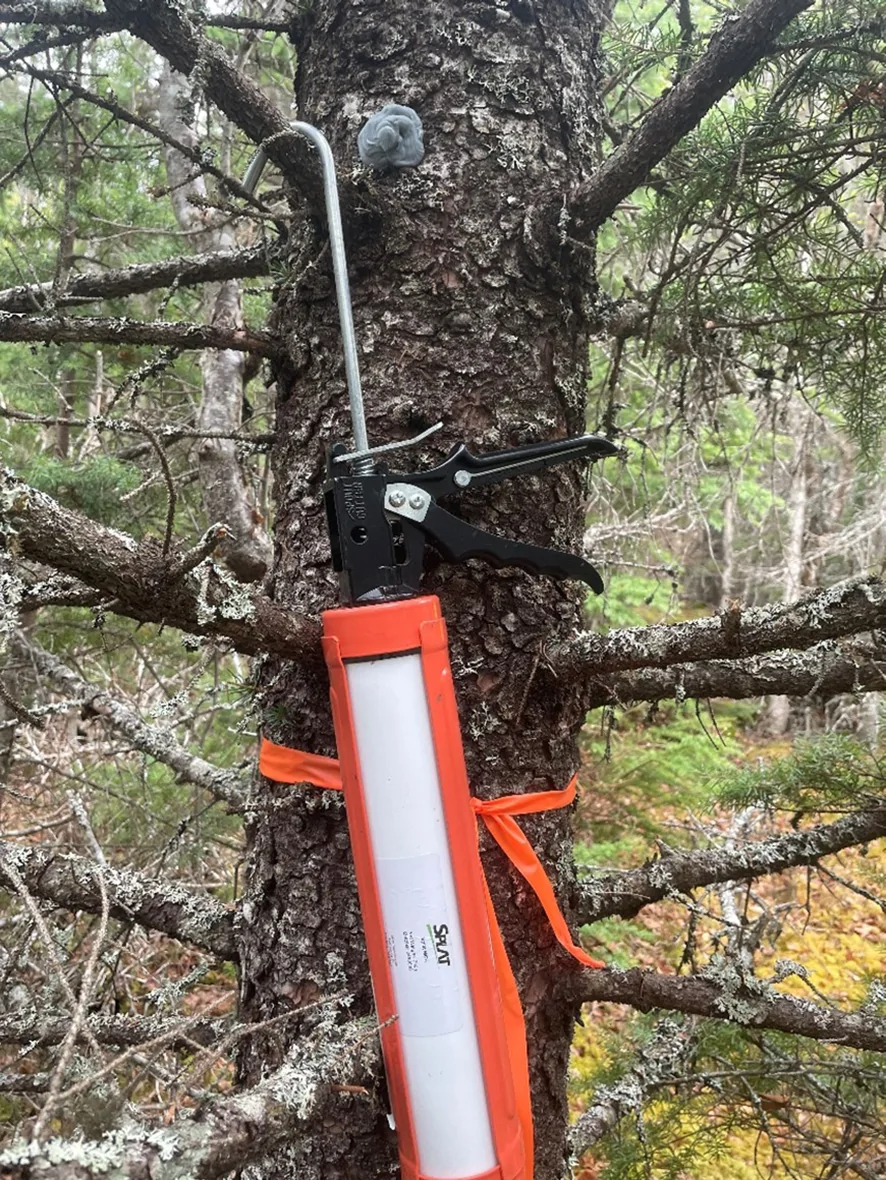
A 17.5-g dollop of SPLAT MCH on the bole of *Picea* x *lutzii* in Alaska, U.S. The size and shape of dollops influence the release of MCH. Photo by C.J. Fettig, USDA Forest Service.

The last 10 years have seen a resurgence in research on *D. rufipennis* due to several large outbreaks (3). Much of this research has focused on the RD&A of inhibitors and was led by the Rocky Mountain Research Station (USDA Forest Service). For example, Hansen et al. (128) combined isophorone and sulcatone with MCH which reduced the number of mass attacked *Pi. engelmannii* on 0.79-ha experimental plots in Utah. Subsequent research converged on an effective blend dubbed *Acer* kairomone blend (“AKB”) that included linalool, β-caryophyllene, and (*Z*)-3-hexen-1-ol combined with MCH based on results from assays conducted in Colorado, New Mexico, Utah, and Wyoming (129, 130). Later, Audley et al. (16, 131, 132) reported SPLAT MCH (**Figure 4**) combined with acetophenone, (*E*)-2-hexen-1-ol, and (*Z*)-2-hexen-1-ol performed as well as SPLAT MCH combined with AKB in Alaska, Colorado, Utah and Wyoming. Recent research evaluating SPLAT MCH has found that MCH alone is more effective for protecting *Picea* from *D*. *rufipennis* than previously suggested (see 123). A study led by the Pacific Southwest Research Station is evaluating the efficacy of SPLAT MCH for protecting 0.41-ha experimental plots of *Pi*. *engelmannii* from *D*. *rufipennis* in Wyoming during a severe outbreak. Preliminary results will be available in 2027.

### 4.6 Ips perturbatus

The host range of northern spruce engraver, *Ips perturbatus* (Eichhoff), includes *Picea*. Preferred hosts are *Pi*. *glauca*, *Pi*. x *lutzii*, and *Pi*. *sitchensis* (133). Males initiate host colonization and release the aggregation pheromones ipsenol, ipsdienol and *cis*-verbenol (134). During the later stages of host colonization, the antiaggregation pheromone verbenone is released by males and females and inhibits attraction to the host tree (134, 135).

Graves et al. (136) found (*E*)-conophthorin and verbenone reduced colonization and mortality of *Pi*. x *lutzii* in south-central Alaska. In two separate assays, levels of tree mortality in the control treatment were 100%, while 0% and 30% mortality occurred in the inhibitor treatment. Conophthorin is produced by birch, *Betula* spp., and other tree species (137, 138), cone and bark beetles (139, 140), and other organisms. Expanding on Graves et al. (136), Fettig et al. (141) demonstrated (*E*)-conophthorin and verbenone reduced colonization of *Pi*. *glauca* slash by *I*. *perturbatus* in Interior Alaska. Natural and anthropogenic (e.g., road building) disturbances produce large quantities of dead and dying *Picea* that serve as ideal hosts for *I*. *perturbatus* (133). If favorable weather coincides with abundant host materials, *I*. *perturbatus* populations may erupt and cause mortality of *Picea* over extensive areas. As such, the research of Fettig et al. (141) has implications for managing *I*. *perturbatus* populations by reducing reproduction in slash and other forest debris. While several formulations of verbenone are registered for tree protection in the U.S., (*E*)-conophthorin is not registered (**Table 2**).

### 4.7 Ips pini

*Ips pini* is among the most widely distributed bark beetle in North America (142). In the western U.S., preferred hosts include *P. ponderosa* and *P. contorta*. Males initiate host colonization and release the aggregation pheromones ipsdienol (26, 143, 144) and lanierone (145). There is notable geographic variation in the enantiomeric ratios of ipsdienol produced by *I*. *pini*. For example, populations in New York, U.S. produce and are attracted to roughly 50:50 ratios of (–)- and (+)-ipsdienol (144) whereas populations in California produce primarily (–)-ipsdienol (26, 146). Lanierone synergizes attraction to ipsdienol except in California (147). Both male and female *I*. *pini* produce verbenone which may function as an antiaggregation pheromone, but the cessation of ipsdienol production by males is likely the main factor terminating aggregation to host trees (56).

Verbenone has been demonstrated to inhibit *I. pini* attraction to baited traps and logs (148, 149). In Arizona, Gaylord et al. (150) evaluated two formulations of verbenone, SPLAT Verb and verbenone pouches, and found both significantly reduced trap catches compared to the control. No differences were observed between SPLAT Verb and the verbenone pouch. In a subsequent trapping assay, acetophenone, (*E*)-2-hexen-1-ol + (*Z*)-2-hexen-1-ol were combined with SPLAT Verb and verbenone pouches, but the addition of these NHVs had no effect on levels of inhibition (151). We are unaware of any assay demonstrating inhibition for tree protection (**Table 2**). A study led by Forest Health Protection (USDA Forest Service) in 2024–2025 evaluated the efficacy of SPLAT Verb for protecting *P*. *ponderosa* from *I*. *pini* in Arizona but was inconclusive due to limited *I*. *pini* activity (C.J.F. et al., unpubl. data).

## 5 Factors that influence the efficacy of inhibitors

### 5.1 Density of the target bark beetle species

While never evaluated in a controlled study, evidence strongly suggests that efficacy declines with increasing population densities of the target bark beetle species (**Table 3**). For example, Progar (152, 153) evaluated verbenone pouches for reducing levels of *P*. *contorta* mortality in Idaho. Verbenone was very effective the first year of the study but efficacy declined thereafter. The author attributed the decline to increases in *D*. *ponderosae* population densities in subsequent years and concomitant decreases in the availability of preferred hosts (i.e., large-diameter *P*. *contorta*). Similarly, Bentz et al. (154) reported reductions in the efficacy of verbenone pouches when >140 P. *contorta* ha^-1^ were infested by *D*. *ponderosae* the previous year. Based on these and other data, authors have suggested that verbenone treatments may not be effective for *D*. *ponderosae* without sanitation (brood tree removal) if >15–20% of *P*. *contorta* are infested with *D*. *ponderosae* at the time of treatment (e.g., 79, 155). This threshold is quite high, for example Audley et al. (4) reported that the highest average annual rates of *P*. *contorta* mortality among states in their study ranged from 5% (Wyoming) to 23% (Utah). Ross (112) argued that the primary reason for the high efficacy observed for MCH and *D*. *pseudotsugae* compared to MCH and *D*. *rufipennis* and verbenone and *D*. *ponderosae* is that the two latter species can reach population densities far beyond those of *D*. *pseudotsugae.* Inhibitors are most effective for suppressing small, localized populations of bark beetles (e.g., 156). Users are encouraged to apply inhibitors during the early stages of an infestation.

**Table 3:** Key factors influencing the efficacy of semiochemical inhibitors for protecting conifers from bark beetles in the western United States.

Factors	Relationship	Effects
Density of the target bark beetle species	Negative	Efficacy declines with increasing population density.
Tree density	Mixed	Lower densities reduce the susceptibility and vulnerability of forests to some bark beetles. Higher densities increase the concentration and directionality of inhibitor plumes due to the effects of tree density on the microclimate of forests.
Variability in responses to volatile stimuli	Mixed	While not well studied, inhibition is affected by genetics, sex, the arrestment response of flying bark beetles, habituation, and the nutritional status of bark beetles, among other factors.
Stereochemistry and chemical stability of inhibitors	Mixed	The enantiomeric composition of verbenone has less effect on inhibition than expected. Concerns of the chemical stability of verbenone in modern release devices are unfounded. There are still questions about the chemical stability of other inhibitors in release devices, and of verbenone and other inhibitors once released into the active airspace of forests.
Release rates	Positive	Higher release rates can result in higher levels of inhibition, but the effect is far from ubiquitous and may not translate to detectible differences in tree protection. Some semiochemicals are multifunctional meaning that they are attractive at low-intermediate concentrations and inhibitory at high concentrations. Concerns regarding multifunctional effects of verbenone on *Dendroctonus ponderosae* are unfounded.
Levels of inhibition	Positive	Higher levels of inhibition result in higher levels of tree protection.
Zone of inhibition	Positive	Higher zones of inhibition result in higher levels of inhibition. However, most inhibitors exert relatively short-range inhibition in the target bark beetle species.
Other stressor and disturbances	Negative	The combined effects of multiple disturbances can further compromise tree health, reducing the efficacy of inhibitors.

### 5.2 Tree density

Tree density can affect the susceptibility and vulnerability of forests to bark beetles (1) and the microclimate within forests, including wind, short-wave radiation and temperature (157). High stand densities increase competition among trees for growing space; reducing defenses and often making trees more susceptible to colonization by bark beetles (**Table 3**). Fewer beetles are required to overcome trees with compromised defenses as photosynthates are allocated to different uses in an order of priorities, with insect and disease resistance mechanisms among the lowest priorities (158). The fewer bark beetles required to kill a tree, the lower the likelihood that inhibitors will be effective as they rarely result in complete inhibition of the target bark beetle species.

Thistle et al. (159) examined near-field canopy dispersion of a tracer gas, as a surrogate for bark beetle pheromones, within the trunk space of trees. They showed that when surface layers of air are stable gas plumes remained concentrated and directional because of the suppression of turbulent mixing. Lower density stands result in unstable layers of air and multi-directional traces that dilute gas concentrations, which could reduce the efficacy of inhibitors for tree protection. Later, Strand et al. (160, 161) modeled surrogate pheromone dispersion under different forest canopy conditions. Their research provides some guidelines for applying inhibitors in forests. For example, for *D*. *pseudotsugae* results suggested that wider spacings of release devices than currently employed could be effective for tree protection if release rates per release device were increased (161).

Differences in stand density and its effects on pheromone plumes likely explain differences in the efficacy of verbenone for *D*. *ponderosae* in *P*. *contorta* forests compared to *D*. *ponderosae* in *P*. *ponderosa* forests. It should be noted that some inhibitors are effective for tree protection in forests of low tree density, as demonstrated for Verbenone Plus and *D*. *brevicomis* and *D*. *barberi* (Sections 4.1, 4.2). To our knowledge, Verbenone Plus has not been evaluated for protecting *P*. *ponderosa* from *D*. *ponderosae*.

### 5.3 Variability in responses to inhibitors

The arrestment response of flying bark beetles to volatile stimuli terminates the response after a period of time (162), presumably for energy conservation. While related research has focused on bark beetle attractants, the arrestment response could explain a lack of response (or reduced responses) to inhibitors by some individuals (**Table 3**). For example, Lindgren et al. (20) reported that habituation was one possible explanation for a decline in the antifeedant effect of verbenone in the European pine weevil, *Hylobius abietis* (L.). Responses may also be affected by the nutritional status of bark beetles (163). Other research suggests that some bark beetles have density-dependent responses to some semiochemicals (164). Furthermore, GC-EAD recordings of bark beetle antennae regularly show disparities in responses to volatile stimuli within and among bark beetle populations and species.

Data on atmospheric concentrations of verbenone in forests in the western U.S. are lacking, although verbenone has been detected (165). Estimates of release from individual *D*. *brevicomis* and *D*. *ponderosae* range from 0–2,800 ng beetle^-1^ (46, 166, 167) to 0–66 ng beetle^-1^ (56, 168), respectively. During outbreaks, relatively large quantities of verbenone are released into forests. Despite this, bark beetles are still able to successfully locate and colonize hosts, suggesting that selection may favor behaviors that help beetles circumvent the gross effects of verbenone during outbreaks compared to other periods of time (79).

### 5.4 Stereochemistry and chemical stability

In bark beetles, substantial amounts of both an enantiomer and its antipode, or *cis-* and *trans*-isomers [(*Z*)- and (*E*)-isomers], may be required to elicit a full behavioral response, whereas in other cases the opposite isomer may be inactive. Verbenone has two enantiomers, (*S*)- (–)- and (*R*)-(+)-, with ratios varying widely within and among bark beetle populations and species (15). Racemic and (–)-verbenone are more inhibitory to *D*. *ponderosae* than (+)-verbenone (74), which although not well studied agrees with data for *D*. *brevicomis* (169). Overall, the enantiomeric composition of verbenone has less effect on inhibition than might be expected (**Table 3**). (–)-Verbenone is most commonly used for tree protection in the western U.S.

Verbenone may be photoisomerized to chrysanthenone which has no effect on bark beetles (170). This was expressed as an early concern for experimental formulations of verbenone, so much so that UV stabilizers were added to the first commercial verbenone pouches. Fettig et al. (49) reported chrysanthenone was undetectable in verbenone pouches that were left in *P*. *ponderosa* forests in California for several weeks. Fettig et al. (94) reported that only after dollops of SPLAT Verb were aged in the field for >12 months were trace amounts of chrysanthenone detected, which is inconsequential as the biological effect of verbenone is desired for only a few months for tree protection. Despite these data, there are still questions about the chemical stability of other inhibitors in release devices, and of verbenone and other inhibitors once released into the active airspace of forests.

### 5.5 Release rates and doses

Inhibitors spread downwind from their point of release in plumes of varying concentrations and directions influenced by forest structure, weather, and other factors. Molecules are detected by olfactory receptors or ionotropic receptors in the antennae and maxillary palps of bark beetles (171). Higher concentrations (i.e., release rates) of inhibitors can result in higher levels of inhibition (e.g., 64, 172, 173), but the effect is far from ubiquitous (174) and may not translate to detectible differences in levels of tree protection (e.g., 131, 132). Byers (175) demonstrated that antennal responses to bark beetle pheromones are best described by kinetic functions which often yield a sigmoidal response curve on a logarithmic scale. Differences in responses are most often observed between release rates with at least an order of magnitude difference. Several authors have speculated that failures in tree protection have resulted from the passive release of inhibitors (e.g., 79), which is largely controlled by temperature for most release devices. However, studies have generally found that commercial release devices are of sufficient dose to maintain adequate release rates under field conditions for *D*. *ponderosae*, *D*. *pseudotsugae*, and *D*. *rufipennis*. In warmer climates, where the target bark beetle species may be active for much of the year (e.g., *D*. *barberi*), more than one application of inhibitors may be necessary (**Table 2**).

Some bark beetle semiochemicals are multifunctional (176) meaning that they are attractive at low-intermediate concentrations and inhibitory at high concentrations. This is frequently expressed as a concern for verbenone as release rates slowly decline over time until the dose of verbenone in release devices is exhausted or the release devices are removed from the field. Based on conversations with colleagues, this concern seems to arise from Experiment 6 in Lindgren and Miller (177; see **Figure 1** on p. 761) which compares the response of *D*. *ponderosae* to six release rates of verbenone (0.0, 0.1, 0.2, 1.8, 3.1, and 12.3 mg d^-1^) in baited traps. While the means reported for 0.1 and 0.2 mg d^-1^ are larger than for 0.0 mg d^-1^ (177), the differences are not statistically significant. To our knowledge, a multifunctional effect has never been demonstrated for verbenone and *D*. *ponderosae* (**Table 3**). While there is evidence of a multifunctional effect for MCH and *D*. *pseudotsugae* (105, 178, 179), which has been challenged by some other researchers (103, 180), to our knowledge this has never been reported as a concern following applications of MCH for tree protection. This is despite MCH being among the most widely used and well-studied of bark beetle inhibitors (Section 4.4).

We stress the importance of properly applying inhibitors in the field, which can affect release rates. For example, bubblecaps, pouches and other release devices should not overlap on the tree bole. For SPLAT Verb and SPLAT MCH, the shape and size of dollops affect release rates. Furthermore, it is important to report both doses and release rates of inhibitors in publications as release rates can vary independent of dose (174). Trapping and tree protection assays evaluating different formulations of an inhibitor should standardize release rates on a unit area^-1^ basis to the extent possible.

### 5.6 Strength of inhibition

Research shows that levels of inhibition vary by inhibitor, bark beetle species, stand and environmental conditions, and the ratio of attractants to inhibitors (e.g., 28, 56, 173, 181). In tree protection assays, the use of baits likely provides a rigorous evaluation of efficacy at the expense of detecting more subtle treatment effects.

### 5.7 Zone of inhibition

Most inhibitors exert relatively short range inhibition in the target bark beetle species (**Table 3**). For example, maximum inhibition (the farthest distance from an inhibitor that the highest levels of inhibition statistically occurred, based on data from baited traps placed at various distances from an inhibitor) of *D*. *barberi* to Verbenone Plus was 3 m in West Texas (64). The maximum inhibition provided by verbenone was 2 m for *D*. *brevicomis* in *P*. *ponderosa* forests in California (49) and ≤4 m for *D. ponderosae* in *P*. *contorta* forests in British Columbia (182) and Utah (94). Maximum inhibition of MCH (and other inhibitors) was 4 m for *D*. *rufipennis* in *Picea* forests in Alaska and 12 m in Colorado (132), and 9 m for *D*. *pseudotsugae* in *Ps*. *menziesii* forests in Idaho (183). These data are useful for informing the spacing of release devices in the field.

### 5.8 Other stressors and disturbances

Bark beetles are just one disturbance that impact forests. The effects of multiple disturbances can be additive or synergistic, influencing overall tree health and the efficacy of inhibitors for tree protection (**Table 3**). For example, fire-injured trees may be more susceptible to bark beetles due to release of host compounds and reductions in host defenses from heating and wounding of tree tissues (184).

## 6 Barriers to the use and availability of inhibitors

### 6.1 Investments

The RD&A of bark beetle inhibitors involves substantial risks and investments, which are barriers to producing products for minor crops, such as forest trees. Costs are exacerbated by the need to work in areas that are remote, rugged, and often difficult to access. Overall, modest investments in research (likely <$0.5 million USD yr^-1^) are made which is surprising given the substantial impacts of bark beetles on forests; for example, some years causing more tree mortality than wildfires (185). In the agricultural sector, costs associated with RD&A of pheromones for pest management yield a benefit-to-cost ratio of $2,655–$26,548 USD to $1 (186). Similar data do not exist for the forestry sector but forests represent greater than a third of the land base in the U.S., and wood products alone contribute ~5% of total U.S. manufacturing gross domestic product (187). The ecosystem goods and services provided by forests are critical to human health and wellbeing and, while very difficult to quantify (188), are likely in the trillions of USD yr^-1^ in the U.S. alone.

Furthermore, investments made in the RD&A of bark beetle inhibitors inform development of inhibitors for other pests. For example, SPLAT Verb was registered in Hawaii, U.S. in 2025 for protecting ‘ōhi‘a, *Metrosideros polymorpha* Gaudich, from ambrosia beetles that vector a fungal disease known as rapid ‘ōhi‘a death (ROD) (189). Since the 2010s, ROD has killed >1 million ‘ōhi‘a, an important keystone species in Hawaii. Researchers have concluded that SPLAT Verb has the potential to minimize the spread of ROD in Hawaii (190–192). In the southeastern U.S., the redbay ambrosia beetle, *Xyleborus glabratus* Eichhoff, vectors a fungal pathogen, *Raffaelea lauricola* T.C. Harr., Fraedrich & Aghayeva, which results in laurel wilt disease. Laurel wilt disease has caused mortality of hundreds of millions of redbay, *Persea borbonia* (L.) Spreng, and other plants. Hughes et al. (193) demonstrated that SPLAT Verb reduces *X. glabratus* attacks on *Pe*. *borbonia* and concluded that SPLAT Verb has potential as part of an integrated management strategy for control of laurel wilt disease. Formulations of bark beetle inhibitors have also been used in several agricultural systems including coffee for coffee berry borer, *Hypothenemus hampei* (Ferrari) (194, 195).

Some barriers can be alleviated by public-private partnerships and incentives (Section 7.4). To that end, recent, new products for *D*. *pseudotsugae* and *D*. *rufipennis* resulted from collaborations among the USDA Forest Service, a state forestry agency, academia, and industry. In response to the *D*. *ponderosae* outbreak in Colorado, the Colorado State Forest Service has coordinated and cost shared verbenone applications with local communities. They estimate ~70,000 verbenone release devices, mostly pouches, will be distributed by the state’s semiochemical program during 2022–2027 (196, 197).

### 6.2 Registration

As discussed, inhibitors are biochemical pesticides that must be registered by the EPA and state regulatory agencies (Sections 2.3, 2.4). There are fees for registration and other costs associated with environmental and human health testing requirements (32).

### 6.3 Managing expectations

Meta-analyses by Schlyter (198) for *D*. *ponderosae* and *I*. *typographus* (L.), the latter a species that causes extensive tree mortality in Europe, based on 9 articles (32 assays) and for global *Dendroctonus* and *Ips* spp. by Afzal et al. (199) based on 52 articles (863 assays) reported substantial negative (i.e., reductions in bark beetle attacks and tree mortality) effects. A reasonable concern with meta-analyses is publication bias, whereby insignificant (or counterintuitive) results may not be published or published in less accessible (gray) literature. However, Schlyter (198) used funnel plots analyses in his study to test for publication bias and found no effect.

There is disagreement among forest health specialists regarding the efficacy of bark beetle inhibitors for tree protection, which has limited more widespread use. Some of this is based on a paucity of data, inconsistent or conflicting results, failures in efficacy, misinterpretations of literature, and/or a lack of understanding of the many factors that affect efficacy for tree protection (Section 5). For example, regarding the latter, Ross et al. (127) reported that MCH was ineffective for protecting *Pi*. *engelmannii* from *D*. *rufipennis*. This led to MCH being considered ineffective for protecting *Picea* from *D*. *rufipennis* for almost two decades. In their study, 1-ha plots were treated along the perimeter of plots with MCH bubblecaps. More recent assays have generally demonstrated strong treatment effects (Section 4.5) based, in part, on the recognition that MCH exerts relatively short-range inhibition in *D*. *rufipennis* (132), which has been addressed by applying higher and more uniformly distributed release points of MCH on individual trees and within *Picea* stands.

A common concern is the inverse relationship between the population density of the target bark beetle species and efficacy of inhibitors (Section 5.1), which is important but affects other suppression tactics (e.g., sanitation and trap trees; 12) with the exception of insecticides. Insecticides, when properly applied, are very effective regardless of beetle population densities but applications of insecticides are limited to small spatial scales (200). Furthermore, many sites (e.g., campgrounds) where insecticides are applied occur near streams limiting applications due to restrictions imposed by no-spray buffers to protect aquatic organisms (201); whereas inhibitors can be applied more widely and with less restrictions.

Potential users should be cognitive of the factors that affect the efficacy of inhibitors for tree protection (Section 5) and the limitations imposed by these factors, and use this information to make informed decisions regarding their use. Assistance is available from the USDA Forest Service, state natural resources agencies, and university Cooperative Extension Service offices.

### 6.4 Nontarget effects

Overall, little is known about the effects of bark beetle inhibitors on nontarget organisms. Based on a study in British Columbia, Lindgren and Miller (202) hypothesized that predators specializing on early successional bark beetles are inhibited by verbenone, while most woodborers and generalist predators do not respond to verbenone or are attracted to verbenone. Fettig et al. (203) found that *Temnoscheila chlorodia* (Mannerheim) (Coleoptera: Trogossitidae), a common bark beetle predator in the western U.S., is attracted to high releases of verbenone in California, and thus verbenone treatments could enhance levels of predation by this species (204). Erbilgin et al. (54) reported that verbenone was inhibitory to *T*. *chlorodia* but that acetophenone had no effect on *T*. *chlorodia*. The valley elderberry longhorn beetle, *Desmocerus californicus dimorphus* Fisher, is a cerambycid endemic to lowland forests of the Central Valley of California that is listed as threatened (205). As a result of verbenone being demonstrated to inhibit other cerambycids (206), we have had an experimental use permit restrict the application of verbenone to mountainous areas in California.

Methylcyclohexanones similar in molecular structure to MCH have been shown to repel bees (Hymenoptera: Apiformes) (207, 208). In fact, some related research originated from a desire to use methylcyclohexanones to repel bees from areas treated with pesticides (209). In forests, wild bees provide important pollination services to understory plants, and some MCH treatments have been challenged by concerned citizens in the western U.S. on the basis of MCH having a potential negative effect on pollinator communities. To address this, Foote et al. (210) evaluated the effects of MCH on wild bee communities in a *Ps*. *menziesii* forest in Idaho and a meadow in Montana. In both studies, there were no differences in bee abundance, bee species richness, or bee species diversity between MCH-treated plots and untreated plots. The authors concluded that treatment of *Ps*. *menziesii* forests with MCH has no effect on bee communities.

There are human health and ecological risk assessments available for applications of MCH (211) and verbenone (212).

## 7 Opportunities

### 7.1 Future RD&A

Perhaps one of the more prominent yet elusive goals in the field of bark beetle inhibition is identification and development of a universal bark beetle inhibitor. Verbenone is considered a universal inhibitor by some authors (15); however, as has been demonstrated for *D. brevicomis* (Section 4.1), verbenone alone is insufficient to impart tree protection in some bark beetle-host systems. The value of a universal inhibitor is obvious as it would drastically reduce the time, cost, and efforts involved in RD&A. A practical opportunity exists for a few, interchangeable combinations of compounds (e.g., combining AKB or acetophenone, (*E*)-2-hexen-1-ol + (*Z*)-2-hexen-1-ol with antiaggregation pheromones) but requires further study. To that end, Verbenone Plus warrants evaluation for protecting *P*. *ponderosa* from *D*. *ponderosae* based on its efficacy for *D*. *brevicomis* and *D*. *barberi* (Sections 4.1, 4.2).

Another opportunity is the vast number of volatile compounds that have not been investigated for bark beetles as much of the focus has been on relatively few compounds (23). It may be that volatile components from ecologically disparate nonhost sources could provide a valuable source of new inhibitors. For example, landing rates of walnut twig beetle, *Pityophthorus juglandis* Blackman, on its host, *Juglans hindsii* (Jeps.) Jeps. ex R.E. Sm., and several nonhost tree species were compared in a riparian forest in California (213, 214). One nonhost tree species evaluated was the non-native red river gum, *Eucalyptus camaldulensis* Dehnh. Significantly fewer *Pit. juglandis* landed on *E. camaldulensis* than on *J. hindsii* even when nonhost branches were within 0.5 m of host branches (213) or when nonhost branches were baited with *Pit. juglandis* aggregation pheromone (214). The apparent avoidance of *E. camaldulensis*, and other nonhost tree species, by *Pit*. *juglandis* in these studies suggests an inhibitory effect by volatile compounds within them. Studies like these may reveal rich sources of compounds previously overlooked for even well-studied bark beetle-host systems.

While this review focuses on the western U.S., similar research has been conducted on important bark beetle species in western Canada (28), the eastern U.S. (204, 215), and Europe (15). Experiences gained and lessons learned in the western U.S. have helped inform the RD&A of bark beetle inhibitors in these and other regions and vice versa. In particular, researchers in the western U.S. and western Canada have worked in close collaboration given the similarity in forest conditions, bark beetle species, and bark beetle impacts (3). Overall, substantial opportunities exist for increasing coordination and collaboration among researchers focused on developing new (or better) inhibitors, inhibitory blends, and formulations.

### 7.2 Automating and optimizing applications

An area worthy of investigation is the development and application of remote deployment technologies. One such strategy could be to modify unmanned aerial vehicles (UAVs) or drones to apply inhibitors in forests; increasing the number of trees or size of areas treated, especially in more remote and inaccessible terrain. Additionally, for some formulations of inhibitors UAVs or drones could allow for applying more release points unit area^-1^ and/or higher release points on the tree bole, which may enhance tree protection and reduce the person-hours required and thus total costs associated with treatments. Potential hurdles include payload limitations, the costs of modifying UAVs or drones, the availability of licensed pilots, maneuverability issues, and limited accessibility to tree boles (e.g., *Picea* typically retain dense lateral branches that may prohibit access). Recent advances in robotics for agriculture (216, 217) could represent the frontier for automating applications of bark beetle inhibitors in some terrains.

Although not studied in the western U.S., UAVs fitted with hyperspectral imaging technologies have promise for identifying infested trees. For example, in Europe the classification accuracy of *Picea* infested with *I*. *typographus*, healthy *Picea*, and dead *Picea* was 76% using UAVs fitted with miniaturized hyperspectral frame imaging sensors operating at wavelengths of 500–900 nm (218). More recent research has focused on early detection of *Picea* infested with *I*. *typographus*, with the earliest occurring about 3 weeks after attacks were initiated (219). Nearby areas of uninfested trees could be prioritized for assessment for treatment with inhibitors.

### 7.3 Developing more efficient means of synthesis

The cost, purity, and availability of certain inhibitors (e.g., (*E*)-conophthorin) is a substantial hurdle for some bark beetle-host systems (28, 220). Synthesis of inhibitors is an obvious chokepoint and improvements could reduce costs and improve availability. For instance, advancements in microbial synthesis of compounds offer potential for reducing costs and improving purity (221). Similarly, Turczel et al. (220) described the application of olefin metathesis reactions to insect pheromone synthesis, including brevicomin and frontalin, as a method for reducing costly reagent catalysts and their associated waste products compared to traditional synthesis pathways. Additional research will likely drive further improvements in synthetic approaches and technologies.

### 7.4 Streamlining regulatory processes and incentivizing investments

Recent decades have seen substantial improvements and advancements in the science, application, and commercial markets for biopesticides in the U.S. (35). While efforts have been made to streamline regulatory processes, opportunities still remain. For example, further reducing variability of regulations among states (and countries) could provide more consistency for biopesticide producers. Another opportunity includes treating similar compounds as registration classes rather than treating each new compound individually. While there can be considerable variability in bark beetle responses to stereoisomers of a given compound (28), there is likely far less variability in threats to environmental and human health. Reviewing and/or regulating broader classes of biopesticides could reduce registration times and costs, and incentivize additional RD&A. Other incentives could be modeled after The Presidential Green Chemistry Challenge Awards (PGCCA), an annual program sponsored by the EPA in partnership with the American Chemical Society that recognizes and promotes innovations in biopesticides (222). Since 1996, PGCCA has recognized 144 technologies that have eliminated >376 million kg of hazardous chemicals annually. EPA’s Small Business Innovation Research (SBIR) Program provides “seed money” to small businesses to develop and commercialize innovative technologies. Phase I awards are provided for up to $100,000 USD while Phase II awards are up to $400,000 to further develop and commercialize promising technologies (223).

### 7.5 Integrating inhibitors with other management tactics

“Push-pull” is a logical integration of inhibitors to “push” target insects from an area and attractants to “pull” target insects into traps where they are captured and killed (17). While push-pull is used in several agricultural systems, it is rarely used for bark beetles (199). Evidence to support the use of push-pull for bark beetles in the western U.S. is lacking but only limited study has occurred (92).

The use of insecticide bole injections to protect trees from bark beetles is increasing in the western U.S. (200). Following injection, the insecticide is transported throughout the tree to the phloem where bark beetles feed. Under optimal conditions this takes several weeks to months, but may take up to one year in high-elevation forests (e.g., for *D*. *rufipennis*). Inhibitors could be used to reduce the risk of injected trees being colonized by bark beetles before adequate concentrations of insecticides have reached the phloem.

To increase the overall resilience of forests to a multitude of stressors and disturbances, efforts are required to restore some forests and to convert highly susceptible forests to less-susceptible forests of more climate-adapted tree species (or mixtures of tree species). The primary tools for this work are thinning, prescribed burning, and planting select tree species and genotypes. Integrating the use of inhibitors with forest thinning and/or prescribed burning has not been studied, but could be important in meeting restoration and resilience goals in some forests. For example, a study in Oregon found short-term increases in tree mortality attributed to bark beetles occurred after thinning was followed by prescribed burning (224). Despite this, the authors argued that the long-term benefits of the thinning and prescribed burning outweighed the short-term increase in tree mortality attributed to bark beetles. The selective application of inhibitors following prescribed burning could reduce undesirable levels of tree mortality and may warrant consideration in some circumstances. Furthermore, a study modeling the drivers of *D. ponderosae* outbreaks in *P. contorta* forests found the diameter of *P. contorta* was the most significant predictor of mortality (24). Large-diameter *P. contorta* could be treated with verbenone to reduce the risk of colonization by *D. ponderosae*, particularly in areas where retention of larger-diameter *P. contorta* is desirable.

## 8 Conclusions

Inhibitors are one tool in a very small toolbox for reducing the impacts of bark beetles on forests in the western U.S. (12). Currently, there are commercial inhibitors for *D*. *ponderosae* in *P*. *albicaulis*, *P*. *contorta*, *P*. *flexilis* and *P*. *lambertiana* (verbenone), *D*. *pseudotsugae* in *Ps*. *menziesii* (MCH), and *D*. *rufipennis* in *Picea* spp. (MCH) (**Table 2**). An effective inhibitor has been developed for *D*. *brevicomis* in *P*. *ponderosa* (Verbenone Plus), which was recently extended to *D*. *barberi* in *P*. *ponderosa* (64), but is not registered by EPA. Continued RD&A and commercialization of Verbenone Plus would give natural resource managers an important tool to limit *P*. *ponderosa* losses from bark beetles. An effective inhibitor was identified for *I*. *perturbatus* (verbenone + (*E*)-conophthorin) but no further RD&A has occurred since 2013 (**Table 2**). There is some disagreement on the overall efficacy of inhibitors, which has limited more widespread availability and use. Some of this is based on misinterpretations of literature and a lack of understanding of the many factors that affect efficacy (**Table 3**). Existing meta-analyses demonstrate substantial negative (i.e., reductions in bark beetle attacks and tree mortality) effects (Section 6.3).

Future opportunities in the RD&A of inhibitors include (a) developing new (or better) inhibitors, inhibitory blends, and formulations, (b) automating (e.g., via unmanned aerial vehicles) and optimizing applications of inhibitors in the field, (c) developing cheaper and more efficient means of synthesizing inhibitors, (d) streamlining regulatory processes and incentivizing RD&A to expedite the commercial availability of products, and (e) integrating inhibitors with other management tactics. One NHV that warrants further study is (*E*)-conophthorin (22, 23), although it has been expensive to synthesize. More research is needed to refine the efficacy of inhibitors, and to determine under what conditions inhibitors are likely to yield the highest efficacy. This is particularly important as the impacts of bark beetles on forests in the western U.S. are projected to increase, challenging forest health specialists and others to limit losses to ecosystem goods and services. Evidence suggests there are favorable benefit-to-cost returns to be realized with additional investments in the RD&A of bark beetle inhibitors.
